# Comparison of effect and safety of phacoemulsification surgery performed by resident and attending physicians

**DOI:** 10.3389/fmed.2024.1401482

**Published:** 2024-08-07

**Authors:** Siteng Wu, Dongwei Yao, Shanshan Hua, Xiangzhe Li, Yan Shi

**Affiliations:** Department of Ophthalmology, The First Affiliated Hospital of Ningbo University, Ningbo, China

**Keywords:** phacoemulsification, cataract surgery, resident, attending, effect, safety

## Abstract

**Aim:**

The objective of this study is to compare the effect and safety of phacoemulsification surgery performed by resident and attending physicians.

**Methods:**

This was a retrospective study. Eyes with cataract who underwent phacoemulsification surgery at the First Affiliated Hospital of Ningbo University between January 2021 and December 2023 were reviewed. All the patients were followed up for at least 12 months and were divided into two groups according to the surgery performer. SPSS was used to analyze data, considering *p* < 0.05 significant.

**Results:**

Overall, 316 patients with cataract in group 1 (surgery performed by resident physician, *n* = 181) and group 2 (surgery performed by attending physician, *n* = 135) were reviewed. There were no statistically significant differences in patient demographics variables and cataract grade between the groups. The resident surgeon used more cumulative dissipate energy (15.00 ± 7.25 vs. 10.83 ± 6.52, *p* < 0.001) and operation time (20.46 ± 5.69 vs. 12.59 ± 4.61 min, *p* < 0.001) to complete the surgery. Also, the ECL in group 1 was higher (14.87 ± 5.00 vs. 10.77 ± 4.46, *p* < 0.001). The eyes had significant visual improvement in both groups postoperatively (*p* < 0.05), but at the 12-month follow-up, eyes in group 2 had better best-corrected visual acuity [0.10 (0.00, 0.22) vs. 0.10 (0.10, 0.22) logMAR, *p* = 0.039]. Except for month 1, the intraocular pressure was no statistical difference in group 1 and group 2 (14.65 ± 2.52 vs. 15.30 ± 2.34 mmHg, *p* = 0.019). Cases in group 1 were more likely to undergo intraoperative and postoperative complications (37 vs. 14, *p* = 0.031), including cornea edema (*p* = 0.025), capsule tear (*p* = 0.044), and posterior capsular opacification (*p* = 0.027).

**Conclusion:**

The effect of phacoemulsification surgery performed by the resident physician is satisfying, but compared to the attending physician, the higher probability of complications should be paid more attention.

## 1 Introduction

Cataract, one of the most common causes of visual loss, presents a growing challenge on a global scale. With the aging population, the prevalence of cataract is on the rise. Surgery is the most effective treatment for cataract, from intracapsular cataract extraction (ICCE) to femtosecond laser-assisted cataract surgery (FLACS), surgery techniques have developed for hundreds of years (1). Although some studies proved a higher rate of complications compared with extracapsular cataract extraction (ECCE) or ICCE techniques, with the advantage of high efficiency and satisfying visual outcome, phacoemulsification still becomes the preferred cataract surgery technique (2).

Based on the learning curve, phacoemulsification presents challenges in mastery, complications like posterior capsule rupture, transient elevated intraocular pressure, and corneal edema are prone to encounter, especially for surgeons with less experience (3). Taravella et al. (4) found nucleus disassembly and removal, cortex removal, and capsulorhexis were quite hard for residents. Hosler et al. (5) reported that compared to attending physicians, resident needs more phacoemulsification operative times and costs. In the past, the vast majority of phacoemulsification operations were performed by attending or higher-level physicians (6). As technology develops, it is possible for residents to learn phacoemulsification efficiently and safely by using the cataract surgery simulator (7). Therefore, more and more experienced resident surgeons are devoted to the technique (8).

Up to now, there has been no study to evaluate the effect and safety of phacoemulsification operated by residents and attending surgeons. The purpose of this study was to compare the effect and safety of phacoemulsification surgery performed by experienced resident and attending physicians, providing data to measure the feasibility and practicality of resident physician operating phacoemulsification surgery.

## 2 Materials and methods

### 2.1 Subjects

This was a retrospective study and has been approved by the Ethics Review Committee of the First Affiliated Hospital of Ningbo University (057RS-YJ01). The study was conducted in accordance with the tenets of the Declaration of Helsinki. The resident received virtual reality simulation training using the HelpMeSee simulator and performed 50 phacoemulsification surgeries with or without the help of other surgeons. The resident passed the evaluation according to The Ophthalmology Surgical Competency Assessment Rubric (OSCAR) and had the ability to perform phacoemulsification independently. The attending physician was a skilled doctor with at least 10 years of experience in phacoemulsification cataract surgery and performed over 3,000 phacoemulsification surgeries. Patients with any of the following criteria were excluded from the study: other diseases that can seriously affect vision, traumatic cataract, congenital cataract, follow-up less than 12 months, and history of previous intraocular surgery. In all, 316 cases of phacoemulsification cataract surgery performed by resident and attending physician from January 2021 through December 2023 were evaluated.

### 2.2 Surgical procedure and follow-up

Standard phacoemulsification cataract surgery was used in each patient, including the following steps: paracentesis and wound construction, capsulorhexis, hydrodissection, nucleus sculpting, nucleus disassembly and removal, cortex removal, intraocular lens (IOL) insertion, ophthalmic viscosurgical device removal, and wound integrity (9). All surgical steps were performed according to the same standard procedure except for the processing of nucleus. The attending surgeon used the phaco chop technique while the resident used the divide and conquer technique (10). The same phacoemulsification machine was used for all surgery.

The following parameters were obtained at preoperative, 1-day, 1-month, 3-month, 6-month, and 12-month postoperatively: best-corrected visual acuity (BCVA), and intraocular pressure (IOP). Patient age, sex, ocular history, follow-up time, intraoperative and postoperative complications such as capsule tear, vitreous loss, cornea edema, posterior capsular opacification (PCO) et al. were also reviewed. Cataract grade including nuclear opacity (NO) and nuclear color (NC) were recorded preoperatively using The Lens Opacities Classification System, version III (LOCS III) (11). Endothelial cell density (ECD), and endothelial cell loss (ECL) calculated as (ECD preoperatively-ECD postoperatively/ECD preoperatively) × 100, were assessed pre and post-operatively at 1 month. Cumulative dissipated energy (CDE), as a value for phaco energy, along with operation time were recorded as intraoperative variables. BCVA was converted into logMAR values and followed the standards: counting fingers = decimal acuity of 0.014 and hand motion = decimal acuity of 0.005 (12).

### 2.3 Statistical analysis

Statistical analysis was performed with SPSS software version 20.0 (SPSS, Inc., Chicago, IL, USA). Continuous variables were reported as mean ± SD, median (25% percentile, 75% percentile) and rage. Frequency distributions and percentages were used for categorical variables. Normality tests and homogeneity of variance analysis are carried out on continuous variables. Comparison of age, NO, NC, ECD, ECL, CDE, operation time, preoperative, and postoperative IOP between the two groups was assessed by independent sample t-test. Mann–Whitney U test was used to compare the follow-up time and BCVA. Categorical variables were analyzed with the Pearson chi-square test or Fisher’s exact test. A *p*-value <0.05 was defined as statistically significant.

## 3 Results

Three hundred sixteen eyes of 316 patients met the inclusion criteria, a total of 1 attending and 1 resident surgeon performed all the phacoemulsification surgeries independently. The resident surgeon completed 181 cases (group 1) and the attending surgeon performed 135 cases (group 2). None of the procedures had to be converted to ECCE or need extra surgery. The mean age of the patients was 60.83 ± 8.10y (42-83y), among them, 65 cases with diabetes and 82 cases with hypertension. After 13.67 ± 2.59 (12–25) months follow-up, the mean BCVA improved from 0.76 ± 0.44 to 0.14 ± 0.14 logMAR postoperatively (*p* < 0.05). There was no statistically significant difference in patient demographics, follow-up time, NO and NC grade between groups (Table 1).

**Table 1:** Demographic variables and follow-up time in Group 1 and Group 2.

	Group 1 (*n* = 181)	Group 2 (*n* = 135)	*t*/*z*/*x*^2^	*p* value
Age, *y*	60.15 ± 7.63	61.75 ± 8.64	−1.710^a^	0.088
Sex (male:female)	71:110	61:74	1.129^c^	0.288
Follow-up time, *m*	12 (12, 14)	12 (12, 15)	−0.914^b^	0.361
Diabetes, *n* (%)	36 (19.89)	29 (21.48)	0.120^c^	0.729
Hypertension, *n* (%)	50 (27.62)	32 (23.70)	0.619^c^	0.432
Cataract grade NO	3.21 ± 0.56	3.31 ± 0.63	−1.594^a^	0.112
Cataract grade NC	2.45 ± 0.61	2.56 ± 0.71	−1.476^a^	0.141
MED, *n* (%)	20 (11.05)	13 (9.63)	0.167^c^	0.683

Group 1, surgery performed by the resident; Group 2, surgery performed by the attending; NO, nuclear opacity; NC, nuclear color; MED, merge other eye diseases; ^a^Independent sample t-test; ^b^Mann–Whitney U test; ^c^Pearson chi-square test.

The median preoperative BCVA in group 1 and group 2 was 0.60 (0.40, 0.96) and 0.70 (0.40, 1.00) logMAR, respectively (*p* = 0.243, Table 2). There were no statistically significant differences in postoperative BCVA between the two groups except at the 12-month follow-up, the median BCVA was better in group 2 than the group 1 [0.10 (0.00, 0.22) vs. 0.10 (0.10, 0.22) logMAR, *p* = 0.039]. In all, the BCVA in 296 cases (93.67%) reached 20/40 or higher at the last follow-up, with 165 cases (91.16%) in group 1 and 131 cases (97.03%) in group 2. Of those that did not achieve a best-corrected visual acuity of 20/40 or better, diabetic retinopathy, cystoid macular edema and postoperative complications were the main reasons. Besides 1 month postoperatively (14.65 ± 2.52 vs. 15.30 ± 2.34 mmHg, *p* = 0.019), no statistically significant differences were found in the IOP between the two groups before and after the surgery (Table 2). 9 patients (2.85%) occurred high IOP that need local IOP lowering medications after surgery, among them, 7 cases in group 1 and 2 cases in group 2. All cases of intraocular pressure ultimately returned to the normal range, and there were no cases had high IOP or glaucoma.

**Table 2:** Intraoperative variables and clinical outcomes in Group 1 and Group 2.

	Group 1 (*n* = 181)	Group 2 (*n* = 135)	*t*/*z*	*p* value
ECD, cell/mm^2^				
Preoperative	2,529.28 ± 390.69	2,456.73 ± 420.69	1.580^a^	0.115
Postoperative	2,155.85 ± 370.13	2,192.69 ± 393.15	−0.852^a^	0.395
ECL, %	14.87 ± 5.00	10.77 ± 4.46	7.544^a^	<0.001
CDE	15.00 ± 7.25	10.83 ± 6.52	5.279^a^	<0.001
Operation time, min	20.46 ± 5.69	12.59 ± 4.61	13.184^a^	<0.001
BCVA, logMAR				
Preoperative	0.60 (0.40, 0.96)	0.70 (0.40, 1.00)	−1.167^b^	0.243
1-day	0.22 (0.10, 0.30)	0.22 (0.10, 0.30)	−0.102^b^	0.919
1-week	0.10 (0.07, 0.22)	0.10 (0.10, 0.22)	−0.473^b^	0.636
1-month	0.10 (0.05, 0.22)	0.10 (0.05, 0.22)	−0.550^b^	0.583
3-month	0.10 (0.10, 0.22)	0.10 (0.00, 0.22)	−0.775^b^	0.438
6-month	0.10 (0.10, 0.22)	0.10 (0.00, 0.22)	−1.634^b^	0.102
12-month	0.10 (0.10, 0.22)	0.10 (0.00, 0.22)	−2.603^b^	0.039
IOP, mmHg				
Preoperative	13.10 ± 3.50	13.38 ± 3.44	−0.709^a^	0.479
1-day	17.05 ± 4.19	17.25 ± 3.42	−0.456^a^	0.649
1-week	15.11 ± 3.04	15.26 ± 2.29	−0.486^a^	0.627
1-month	14.65 ± 2.52	15.30 ± 2.34	−2.356^a^	0.019
3-month	14.63 ± 2.20	15.09 ± 2.21	−1.845^a^	0.066
6-month	14.78 ± 2.41	15.00 ± 2.05	−0.878^a^	0.381
12-month	15.19 ± 2.56	15.39 ± 2.09	−0.744^a^	0.457

Group 1, surgery performed by the resident; Group 2, surgery performed by the attending; ECD, endothelial cell density; CDE, cumulative dissipate energy; ECL, endothelial cell loss; BCVA, best-corrected visual acuity; IOP, intraocular pressure; ^a^Independent sample t-test; ^b^Mann–Whitney U test.

The ECD between the groups was not statistically different. The resident surgeon used more CDE (15.00 ± 7.25 vs. 10.83 ± 6.52, *p* < 0.001) and operation time (20.46 ± 5.69 vs. 12.59 ± 4.61 min, *p* < 0.001) to complete the surgery. Also, the ECL in group 1 was higher (14.87 ± 5.00 vs. 10.77 ± 4.46, *p* < 0.001). 51 (16.14%) patients had complications of varying severity, 12 (6.62%) cases in group 1 had serious complications including lens cortex residual, capsule tear, or vitreous loss, while only 1 (0.74%) case in group 2 had undergone capsule tear, no statistically significant difference was found (*p* = 0.018, Table 3). There were no cases of dropped nucleus in both group. Intraoperative and postoperative complications were observed in 37 (20.44%) cases performed by the residents and 14 (10.37%) cases performed by the attending physician (*p* = 0.031). Early complications such as cornea edema in group 1 were more than the cases in group 2 (24 vs. 7, *p* = 0.025). Cases completed by the resident surgeon were more likely to have PCO in long-term follow-ups (27 vs. 9, *p* = 0.027). Capsule tear was also more likely to occur in patients performed by the resident (6 vs. 0, *p* = 0.044). Anterior vitreous loss occurred in 5 cases, it was managed by trimming the anterior vitreous without the need for additional complex vitreoretinal surgery. Two cases underwent lens cortex residual in group 1, and the last BCVA of them reached 0.6 and 0.8, respectively. No cases required a second or complex vitreoretinal surgery.

**Table 3:** Complications in Group 1 and Group 2.

	Group 1 (*n* = 181)	Group 2 (*n* = 135)	*x* ^2^	*p* value
Total complications, *n* (%)	37 (20.44)	14 (10.37)	4.675	0.031
Cornea edema, *n* (%)	24 (13.26)	7 (5.19)	5.046	0.025
High IOP, *n* (%)	7 (3.87)	2 (1.48)		0.315
PCO, *n* (%)	27 (14.92)	9 (6.67)	4.913	0.027
Serious complications	12 (6.62)	1 (0.74)		0.018
Lens cortex residual, *n* (%)	2 (1.10)	0 (0.00)		0.513
Capsule tear, *n* (%)	6 (3.31)	0 (0.00)		0.044
Vitreous loss, *n* (%)	4 (2.21)	1 (0.74)		0.407

Group 1, surgery performed by the resident; Group 2, surgery performed by the attending; IOP, intraocular pressure; PCO, posterior capsular opacification.

## 4 Discussion

In comparison to other countries, China has the largest elderly population worldwide, the avoidable blindness like cataract is becoming a troubling challenge. However, the Cataract Surgical Rate (CSR) in China is far lower than the developed countries (13), the per million people CSR only reached 2,205 (8), which is far below demand. This underscores the increasing need for more cataract surgeons in the near future. But cataract surgeries are not easy to have a good grasp., especially for those without systematic training surgeons. Kaplowitz et al. (14) pointed to mastering phacoemulsification surgery skills, a minimum of 70 operations are needed for inexperienced surgeons. Different reports have indicated complications were more likely to happen for the surgeries performed by the residents (15, 16). Teaching operation skills is a time-consuming process, especially with a patient that is awake and under the microscope. Fortunately, as the technique developed, virtual reality cataract surgery simulation came out, which can greatly reduce the number of surgeries, time, and cost required to master cataract surgery techniques (7, 17). Montrisuksirikun et al. (18) suggested after simulation training, surgery complications were less likely to happen and the operation would be completed faster and safer.

In our study, the resident was systematically trained for phacoemulsification surgery, including wet laboratory and visual reality surgery simulation learning using the HelpMeSee simulator. Although in the beginning, the resident probably required help in certain surgery procedures, after performing 50 phacoemulsification surgeries, the resident was skilled enough to get a satisfying score in OSCAR evaluation and could complete the surgery independently. Because of the limitations in retrospective research, some patients were lost to follow-up, we observed 316 patients in all. The attending physician performed more complex cataract surgeries or combined vitreoretinal surgeries, which did not meet our inclusion criteria. So only 135 cases were performed by the attending doctor in this study. The visual outcome was satisfying, the BCVA of cases performed by the resident improved from 0.60 (0.40, 0.96) to 0.10 (0.10, 0.22) logMAR, with 165 cases (91.16%) reached 20/40 or higher at the last follow-up, no statistically significant difference was found compared to cases completed by the attending physician. This also confirms previous research findings that the improvement in vision acuity after cataract surgery performed by the resident is desirable (19).

Many factors such as age, different types of cataracts, and ocular conditions et al. can affect the effectiveness of surgery (19). To this end, we exclude cases with traumatic cataract, congenital cataract, or other diseases that can seriously affect vision to keep the confounding factors as few as possible. We also avoid including complex and high-risk cataract surgeries in research to optimize the outcomes of surgeries. No statistical difference was found in patient demographics, follow-up time, cataract grade, ECD, and preoperative BCVA in two groups, which makes the results more convincing. Some studies researched the outcome and complication rate of cataract surgeries performed by the resident (15, 16, 20), but to the best of our knowledge, this is the first study in China to compare the efficacy and safety of the resident surgeon trained in virtual reality surgery simulation with the attending surgeon in phacoemulsification. Besides, no study compared the IOP status after surgery performed by the resident or attending physicians. Nucleus and cortex incomplete removal can affect the visual outcome and increase intraocular pressure, which are also the hardest steps for cataract surgery (4). So we take IOP into consideration, 7 patients in group 1 and 2 patients in group 2 needed topical antiglaucoma medications, the difference in IOP between the two groups was not statistically significant.

Phacoemulsification surgery is challenging work with many serious complications could occur, any of which could cause damage to the visual acuity or even surgery failure (21, 22). The overall rate of complications was reported from 5 to 37% previously (23–25), in this study, we found 16.10% of cases encountered different degrees of complications, including early postoperative complications like cornea edema and late postoperative complications such as PCO. The major serious complications including lens cortex residual, capsule tear, and vitreous prolapse were more likely to happen for those operations performed by the resident (6.62% vs. 0.74%), slightly lower than previous studies in total (15, 23–25), we thought the resident with experience in visual reality cataract simulator training can explain the difference. We found more patients had cornea edema in group 1, and the difference was statistically significant. We believe this may related to the higher energy used and longer duration of phacoemulsification when surgery was performed by the resident, as well as the instrument was more likely to touch the corneal endothelium during the operation process. Wong et al. (10) proved a significant advantage of the phaco chop over the divide and conquer technique in phaco power and duration. It is the direction that residents can devote to in future phacoemulsification surgeries. Although all the cornea edema was relieved in 2 weeks and did not influence the final visual outcome, some patients still complained about poor visual improvement in the early postoperative period. It is quite necessary to have good propaganda and education before the surgery. PCO is a multifactorial common complication in the late-term follow-up after cataract surgery (26, 27). In our study, patients in group 1 were more likely to have PCO, which may explain the worse visual outcome at the 12-month follow-up. We consider the incomplete polishing of the posterior capsule by the resident physician during surgery may be the cause of this phenomenon. More cases had a vitreous loss in group 1, though the difference was not statistically significant, more attention should be paid to decreasing capsule tear and vitreous prolapse.

Hosler et al. (5) indicated the resident physician takes an average of 12 min longer per eye compared to the attending surgeon to complete phacoemulsification surgery, which is similar to our finding. Capsulorhexis, nucleus removal, and cortex removal were the hardest part for residents during the operation, and more time was needed (4). Regretfully, due to the limit of retrospective study, the time cost of different surgery steps was not recorded and we could not compare this indicator between groups. Meanwhile, a questionnaire could also be designed to obtain subjective feelings of resident physicians about the difficulty of different steps. For future prospective studies, we are looking forward to using the Najjar-Awwad risk score (28) to evaluate the risk of patients undergoing phacoemulsification surgery, to ensure the preoperative eye condition is as similar as possible, and to make sure the surgery is safer for resident physicians to perform. In addition, longer follow-up is needed to compare the long-term effects of surgery.

To conclude, we can state that the effect of phacoemulsification surgery performed by the resident physician is satisfying, but compared to the attending physician, the higher probability of complications should be paid more attention.

## Data availability statement

The original contributions presented in the study are included in the article/supplementary material, further inquiries can be directed to the corresponding author.

## Ethics statement

The studies involving humans were approved by the Ethics Review Committee of the First Affiliated Hospital of Ningbo University (057RS-YJ01). The studies were conducted in accordance with the local legislation and institutional requirements. Written informed consent for participation was not required from the participants or the participants’ legal guardians/next of kin in accordance with the national legislation and institutional requirements. Written informed consent was obtained from the individual(s) for the publication of any potentially identifiable images or data included in this article.

## Author contributions

SW: Data curation, Formal analysis, Investigation, Writing – original draft, Writing – review & editing. DY: Data curation, Methodology, Writing – review & editing. SH: Data curation, Investigation, Writing – review & editing. XL: Conceptualization, Investigation, Writing – review & editing. YS: Conceptualization, Funding acquisition, Writing – review & editing.
